# New Series of the Journal

**Published:** 1850-10

**Authors:** 


### EDITORIAL DEPARTMENT.

### NEW SERIES OF THE JOURNAL.

With the completion of the tenth volume, the connection of the Journal with the American Society of Dental Surgeons terminated, and from the issue of the present number, the publication must be considered an individual enterprize, depending for pecuniary support upon its subscription only, and for its literary maintenance upon the exertions of the editor, and of the able friends who have kindly promised their assistance.

Heretofore, the proper revenue of the Journal has not been adequate to its support, and the annual deficiency has been met by a contribution from the association under whose auspices it was conducted. Without such aid, the publication must have been discontinued long since.

No one acquainted with the matter, can doubt that the Journal has contributed, in a very eminent degree, to the advancement of the science and art of Dental Surgery. For the last ten years, its pages have been communicating to its readers whatever discoveries, inventions or improvements have been made in this branch of surgery. For a considerable time, it was the only vehicle for this intelligence—the only receptacle into which scattered facts or perishable suggestions could be gathered and preserved for the common benefit of the profession.

Since the commencement of the publication of the Journal, six other dental periodicals, two in Europe and four in the United States, have been issued. Of these, only three are now published: the New York Dental Recorder, the Dental Register of the West, and the Dental News Letter.

Each of these periodicals received from the Journal a hearty welcome, and the publication of one after another, was hailed as a good omen, an indication that the members of the profession were becoming aroused to the importance of a higher standard of education, and that the resources of dental surgery were becoming more and more developed, to meet the increasing demand constantly made upon them. It is a cause of regret to us, that any one of these journals has been permitted to languish and expire for want of patronage.

The suggestion of a periodical devoted to the interests of dental surgery, originated with us, and it has been our fortune to be connected editorially with this Journal from its commencement. Formerly, however, we were not alone; others shared the responsibility and labor of editorship. Now we are thrown upon our own resources and the generous assistance of our friends.

Most sincerely do we feel the loss of the valuable advice and aid of those who were formerly associated with us; but we have reason to hope for their assistance, and to expect that the pages of the Journal will frequently be enriched with contributions from their pens.

That we shall be able to please all, is more than we can reasonably expect. In a profession composed of men who differ widely in scientific attainments and practical skill, as well as in opinions with regard to etiquette and the proper standard of theoretical and practical requirement for the profession of dentistry, it is scarcely possible to pursue a course approved by all. We must therefore do the best we can. Advocating fearlessly, and to the utmost of our ability, what we may conceive to be right in theory and practice, we will endeavor to pursue an honest, firm, yet modest course, doing nothing but what is right, and submitting to nothing that is wrong.

We wish it to be understood, that, as editor, while we wish to be courteous to all, we must be independent of all, so far as the management of the Journal is concerned. We will never compromise our honor to gratify any man, nor countenance empiricism in any form or disguise in which it may appear.

In thus frankly declaring our purpose, we do not wish it to be understood that we shall denounce those who may hold opinions adverse to our own. We will interfere with no man's privilege of thought. The pages of the Journal shall always be open to well written articles upon any subjects connected with the interests of the profession, or relating, directly or indirectly, to the science and art of dental surgery. It is manifest, however, that the value of a periodical devoted to the advancement of any department of knowledge, must depend, in a great degree, upon the number and ability of its contributors, and our Journal can never attain the high practical and scientific character which we propose, unless we are cordially aided by the profession. A number of our professional friends have already promised us their aid, and we solicit the co-operation of all who feel any interest in the progress of this branch of medicine.

Our readers will pardon us for mentioning another indispensable requisite for success. It is the promptness of our patrons in the payment of their subscriptions. Publishers of periodicals regard this as a cardinal virtue, and nothing so wonderfully increases their favorable opinion of their subscribers. From past experience, we are sensibly aware of the great inconvenience, amounting often to serious embarrassment, which may be caused by the thoughtlessness of subscribers in not punctually paying the small sums due by them. It is but just, however, to say, that many of the subscribers of the Journal have been very commendably punctual in this respect. From these we hope never to part, and of those who have heretofore been remiss, we have nothing more harsh to say, than to express the hope that they will be more thoughtful for the future.

